# Impact of a virtual coaching program for women physicians on burnout, fulfillment, and self-valuation

**DOI:** 10.1186/s40359-024-01763-0

**Published:** 2024-06-05

**Authors:** Sunny Smith, Nicole Goldhaber, Kathryn Maysent, Ursula Lang, Michelle Daniel, Christopher Longhurst

**Affiliations:** 1 Empowering Women Physicians, 4653 Carmel Mountain Rd. #308-201, San Diego, CA 92130 USA; 2 Department of Surgery, University of California, San Diego, USA; 3 Office of the Chief Medical Officer, University of California, San Diego, USA; 4 Department of Pathology, University of California, San Francisco, USA; 5 Department of Emergency Medicine, University of California, San Diego, USA

**Keywords:** (3) physician coaching, Burnout, Professional fulfillment, Compassion, Moral injury

## Abstract

**Background:**

Coaching has been demonstrated to be an effective physician wellness intervention. However, this evidence-based intervention has not yet been widely adopted in the physician community. Documentation and implementation research of interventions to address physician burnout in real world settings is much needed.

**Objective:**

Assess the impact of a virtual physician coaching program in women physicians.

**Design:**

Pre- and post-intervention surveys administered to participants enrolled in the program (*N* = 329). Effect size was calculated comparing pre- and post-intervention paired data (*N* = 201).

**Participants:**

201 women physicians from 40 states in the United States of America and 3 international participants.

**Interventions:**

Participants were given access to an 8 week virtual coaching program including eight individual, six small group, and 24 large group sessions.

**Main measures:**

Stanford Professional Fulfillment Inventory (PFI) containing categories for assessing professional fulfillment, burnout, and the Clinician Self-Valuation (SV) Scale (a measure of self-compassion).

**Key results:**

Burnout was found in 77.1% (*N* = 155) of participants at baseline, which reduced to 33.3% (*N* = 67) at completion with large effect size (Cohen’s *d* 1.11). The percentage of participants who endorsed significant professional fulfillment started at 27.4% (*N* = 55) and improved to 68.2% (*N* = 137) with a large effect size (Cohen’s *d* 0.95). Self-valuation improved from 17.9% (*N* = 36) of the participants endorsing a compassionate self-improvement perspective to 64% of the same participants eight weeks later. The self-valuation metric showed a very large effect size (Cohen’s *d* 1.28).

**Conclusions:**

Virtual physician coaching programs led by physician coaches can decrease burnout, improve professional fulfillment, and increase self-compassion. Non-institution-based opportunities for coaching available to any physician across the United States and internationally can facilitate access to effective physician well-being interventions.

**Supplementary Information:**

The online version contains supplementary material available at 10.1186/s40359-024-01763-0.

## Introduction

Physician well-being and distress are major concerns in healthcare. The additional stress of the COVID-19 pandemic exacerbated underlying challenges and female physicians have been disproportionately impacted.

Burnout is characterized by emotional exhaustion, depersonalization and perceived lack of accomplishment [1]. It is widespread across medical specialties and career stages, and worsening in recent years [2]. Burnout is associated with medical errors, self-reported suboptimal patient care, reduced professional work effort, self-reported unprofessional behaviors, and lower productivity, demonstrating its ripple effect on patients [3–9]. The COVID-19 pandemic placed additional stress on healthcare workers, and physician burnout rates increased dramatically to an all-time high of 63% [2]. Women physicians have higher reported rates of burnout compared to their male colleagues [10–14]. The current level of physician burnout actively threatens the health of physicians, patients, and society. Interventions to address physician distress, burnout, and moral injury are urgently needed.

The impact of interventions, in increasing order, for burnout currently being implemented in our healthcare system includes mindfulness practices, peer-support groups, coaching and system level intervention [15]. System-level changes are essential, yet we cannot wait solely for the systems to change while the level of distress is so high [16]. Individuals who are suffering need interventions that can bring immediate improvement.

A recent review of physician coaching interventions reveals that each study that met criteria for review resulted in improvement in physician well-being [17]. Initial studies of physician coaching utilize certified coaches who were not physicians. As more physicians have been coached and seen the benefit of coaching in their own lives, an increasing number of physicians have decided to train and become certified coaches themselves. There are currently at least 400 physicians who have trained as coaches and this number continues to increase rapidly [18]. There is an emerging literature documenting the benefit of having physician coaches to coach other physicians [19]. 

The medical culture results in a unique lived experience and understanding of the training process, the hidden curriculum, the demands of the profession, taking call, charting, supervising, patient care, billing, and the current medical practice environment. This results in an immersion of physicians in a culture of self-sacrifice that has allowed the system to continue to ask more of us beyond what most professions would allow or tolerate [20]. Even within the medical profession, physicians experience the health care system differently than physician assistants, nurse practitioners, nurses, medical assistants, and other medical staff [21]. Physician coaches offer a unique opportunity for physicians to be coached by other physicians who are not only certified in coaching methodologies, but also have an intimate firsthand understanding of the demands of the medical culture and medical practices.

Many physicians have delayed seeking support due to concerns about stigma [22]. Concerns include that seeking help may result in a diagnosis that could end up on their medical record and require disclosure for insurance, disability, or state medical licensure. While many states are proactively making changes to their license applications, the concern of physicians seeking support is still present [23]. Given emerging data that coaching is effective at addressing physician well-being, physicians have started to seek out commercially available coaching programs.

It is important to note that coaching differs from therapy in that it does not diagnose or treat mental illness. Coaching assumes that the client is whole and helps them increase self-awareness, access their strengths, utilize tools of metacognition, and recognize and utilize their agency, autonomy, and self-efficacy. Coaching normalizes the full lived human experience of being a physician and allows the client to bring up anything they want to work on in their personal or professional life in a safe, non-judgmental space. Physicians can be referred to therapy or psychiatry if the needs of the physician exceed the scope of coaching. Coaching, therapy, and psychiatry can be used simultaneously, especially considering that burnout and depression frequently co-occur. Any help-seeking behavior typically begets normalization of and encouragement of additional help-seeking behavior as needed and as appropriate titrating up and down depending on varying individual needs over time.

Coaching focuses on self-awareness and helps clients identify their strengths and internal resources to make empowered choices [24]. Coaching interventions designed for physicians have been shown to reduce emotional exhaustion, improve quality of life and well-being, and reduce overall burnout [16, 17, 25]. Previous studies of physician coaching have been performed inside institutions or controlled research studies, but there has been a lack of widespread implementation of physician coaching that can be accessed by individual physicians irrespective of their workplace or geographic location. In addition, further exploration of the effectiveness of different types of coaching programs and programs aimed at specific populations is warranted.

While coaching is relatively common in some fields such as among business executives and professional athletes, real world implementation of coaching for physicians is relatively sparse despite a growing body of evidence for the effectiveness of this intervention. There is not only a moral case but also a business case and a public health case for prioritizing and addressing physician wellness [15, 26]. Despite mounting data from clinical trials, the logistics and access of broad implementation of this intervention remains unknown. Implementation science is the field of bringing research findings to the community and assessing the efficacy in the real world. To the authors knowledge there have been no studies attempting to examine the efficacy of broader implementation of physician coaching in the real world across specialties, locations, and practice throughout the United States. The RE-AIM framework is commonly used in public health issues to assess the translation of scientific advances from research studies into practice [27]. This framework has been utilized to assess dissemination and broader implementation of research findings to population-based impact.

Current access to coaching for physicians may be highly variable depending on the workplace. Some institutions offer access to coaches through the health care institution and there are a growing number of physician coaches and independently run physician coaching companies. These coaching companies offer a wide range of interventions from individual coaching to group coaching and a mix of both.-In some cases, physicians are able to advocate for their workplace to help fund support for coaching. Continuing Medical Education (CME) funds can be utilized for some physician well-being programs including coaching and can be utilized to help make coaching more accessible to individual physicians. Utilizing CME hours and funds for physician well-being may also help physicians choose to access support as it fits within a mental construct that is already familiar and normalized for physicians without any associated pathologizing or stigma.

Past research on the effectiveness of coaching has largely focused on programs implemented within a single institution. Some physicians are not comfortable speaking with coaches from inside an organization due to potential lack of privacy or conflict of interest. Coaching programs that are intentionally separated from and not associated with any workplace may confer benefits and an added sense of safety over institutional programs [16, 28]. 

In this study, we examined the impact of supporting physicians with personal and professional coaching through non-institution-based access. Individual physicians self-selected to participate in an independently run commercially available coaching program designed for women physicians by women physicians who are also certified coaches. Enrollment was widely available to any MD or DO, regardless of specialty, practice type, or practice location.

While the benefits of institutional coaching programs have been recently documented, non-institutional programs have largely been unexamined. Here we begin to address this by exploring the effects of independent coaching specially for women physicians, with the intervention taking place during the initial years of the COVID-19 pandemic.

## Methods

Participants self-selected to enroll in an eight-week intensive virtual coaching program designed to help women physicians (*N* = 329). All physicians who participated in the EWP coaching program were sent pre- post- intervention surveys. The results are based on a program evaluation, and retrospective IRB exception was acquired. The program is commercially available, not centralized to a single academic institution and is run by physicians who are also coaches certified through The Life Coach School. Paired pre- and post-survey participant data from a virtual coaching program evaluation, delivered June 2020 to November 2021 was included in this analysis and the retrospective study was approved by the University of California San Diego Institutional Review Board.

The specific coaching program referenced in this study is delivered by Empowering Women Physicians (EWP) and was conducted entirely remotely during the pandemic via zoom. This program incorporates eight private one-on-one coaching sessions, as well group coaching available at least 3 times per week over eight weeks (Supplementary material). Participants could schedule private one on one sessions any time seven days per week. These live one on one coaching sessions were private between the client and the coach and were not recorded. Longitudinal small group sessions of approximately ten people were conducted weekly in “meeting” mode on zoom so that all participants could see and could get to know one another. These small group sessions were also not recorded. Larger group sessions of varying size were delivered “webinar” style on zoom, with participants able to raise their virtual hands and be coached in any given session or to remain off screen and observe and listen in to see how this coaching may also apply to their own lives. The large group sessions are recorded and made available on replay in a password protected web portal accessible 24 h 7 days a week to coaching program participants. Non-disclosure agreements were in place for privacy. The option for CME credit was offered to participants. There is an option for additional longitudinal participation in a continuity coaching program past the first eight weeks, which is not included in this study.

As a routine part of the coaching program evaluation for CME activity and continual quality improvement, participants completed web-based pre- and post- intervention surveys. As part of the survey, participants completed validated measures of both positive and negative factors related to well-being: the Stanford Professional Fulfillment Inventory (PFI) containing items on fulfillment and burnout, and the Clinician Self-Valuation (SV) tool for measuring self-compassion [29, 30]. These instruments were selected due to their sensitivity to change, which allows evaluation of pre- and post- physician wellness interventions lasting weeks to months. The PFI measures not only burnout but also professional fulfillment. Responses were recorded on five-point Likert scales across survey instruments.

To determine the presence and magnitude of the effect of completion of the coaching program on participants’ fulfillment, burnout and self-compassion survey data was examined using descriptive statistics, 2 tailed t-tests, and chi-squared tests using Excel version 16.55 (Microsoft). A p-value of 0.05 was considered statistically significant.

Implementation of this program into practice in the community was reviewed using reach, effectiveness, adoption, implementation, and maintenance (RE-AIM) framework assessment.

## Results

**Fig. 1:**
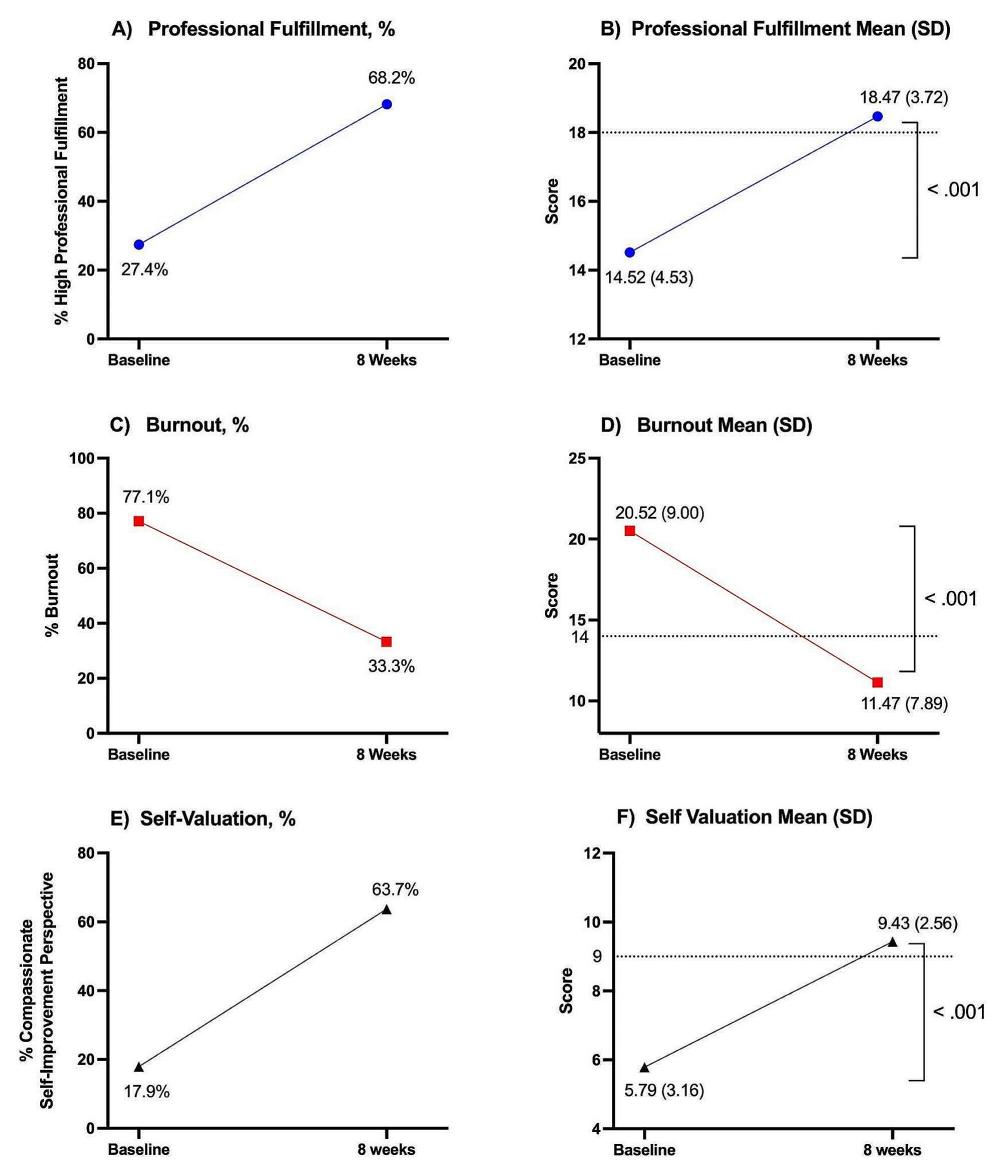
Cumulative results of 201 participants before the start of the program (Baseline) and after completion of the program (8 weeks) are represented. The dotted lines represent the threshold score for each metric and the standard deviation is in parentheses. **(A)** Percentage of participants scoring above the threshold for high professional fulfillment (≥ 18). **(B)** Professional fulfillment mean scores (*p* < 0.001). **(C)** Percentage of participants with scores indicating a high likelihood of experiencing burnout (≥ 14). **(D)** Burnout mean scores (*p* < 0.001). **(E)** Percentage of participants with a compassionate self-improvement perspective (≥ 9). **(F)** Self Valuation mean scores (*p* < 0.001)

The number of participants who completed both the pre- and post- intervention survey resulted in a response rate of 61.1% (201/329).This included women from 40 states across the United States and 3 international participants.  Pre- and post-intervention professional fulfillment, burnout, and self-valuation results can be seen in Table 1 and Figure 1.

**Table 1:** Pre / post intervention measures of an 8-week virtual coaching program for women physicians using the Stanford Professional Fulfillment Inventory (PFI) containing categories for assessing professional fulfillment and burnout, and the Clinician Self-Valuation (SV) Scale. June 2020 – November 2021. *N* = 201

Item	Pre- intervention *n* (%)	Post-intervention *n* (%)	*P* value	Effect Size (Cohen’s d)
**Professional fulfillment** ^**a**^	55 (27.4%)	137 (68.2%)	< 0.0001	0.95
**Burnout** ^**b**^	155 (77.1%)	67 (33.3%)	< 0.0001	1.11
**Self-valuation (Compassionate self-improvement perspective)** ^**c**^	36 (17.9%)	128 (63.7%)	< 0.0001	1.27
	Pre-intervention mean (SD)	Post-intervention mean (SD)	P value	
**Professional fulfillment** ^**a**^ ***-sum of all 6 items below in this scale***	**14.52 (4.53)**	**18.47 (3.72)**	**< 0.0001**	**0.95**
I feel happy at work	2.18 (0.86)	2.86 (0.74)	< 0.0001	0.84
I feel worthwhile at work	2.60 (0.94)	3.24 (0.75)	< 0.0001	0.76
My work is satisfying to me	2.37 (0.93)	3.05 (0.79)	< 0.0001	0.79
I feel in control when dealing with difficult problems at work	2.04 (1.05)	2.92 (0.87)	< 0.0001	0.91
My work is meaningful to me	2.97 (0.96)	3.34 (0.75)	< 0.0001	0.44
I’m contributing professionally (e.g. patient care, teaching, research, leadership) in the ways I value most	2.35 (0.99)	3.06 (0.90)	< 0.0001	0.75
**Burnout** ^**b**^ ***-sum of all items below in this scale***	**20.52 (9.00)**	**11.14 (7.89)**	< 0.0001	1.11
A sense of dread when I think about work I have to do	2.57 (1.20)	1.57 (1.08)	< 0.0001	0.88
Physically exhausted at work	2.49 (1.24)	1.50 (1.16)	< 0.0001	0.83
Lacking in enthusiasm at work	2.41 (1.12)	1.39 (1.14)	< 0.0001	0.90
Emotionally exhausted at work	2.61 (1.18)	1.42 (1.11)	< 0.0001	1.04
Less empathetic with my patients	1.60 (1.20)	0.86 (0.95)	< 0.0001	0.70
Less empathetic with my colleagues	1.76 (1.17)	0.96 (0.98)	< 0.0001	0.75
Less sensitive to others’ feelings/emotions	1.65 (1.12)	0.81 (0.90)	< 0.0001	0.83
Less interested in talking with my patients	1.73 (1.26)	0.86 (1.00)	< 0.0001	0.77
Less connected with my patients	1.70 (1.24)	0.81 (0.93)	< 0.0001	0.82
Less connected with my colleagues	2.00 (1.21)	0.98 (1.02)	< 0.0001	0.92
**Self-valuation (Compassionate self improvement perspective)** ^**c**^ ***-sum of all items below in this scale***	**5.79 (3.16)**	**9.43 (2.56)**	**< 0.0001**	**1.28**
When I made a mistake, I felt more self-condemnation than self-encouragement to learn from the experience	1.50 (1.01)	2.46 (0.75)	< 0.0001	1.08
I was less compassionate with myself than I was with others	1.10 (0.80)	2.07 (0.84)	< 0.0001	1.18
I put off taking care of my own health due to time pressure	1.39 (1.05)	2.30 (0.93)	< 0.0001	0.91
Taking care of my needs seemed incompatible with taking care of my patients’ needs	1.79 (1.06)	2.61 (0.88)	< 0.0001	0.84

^a^If score ≥ 18, the respondent is likely experiencing high professional fulfillment

^b^If score ≥ 14, the respondent is likely to be experiencing burnout

^c^If score ≥ 9, the respondent has a compassionate self-improvement perspective

### Professional fulfillment

The mean professional fulfillment score increased from 14.52 (SD 4.5) to 18.47 (SD 3.7), (*p* < 0.001). A professional fulfillment score of 18 or higher using the Stanford PFI on this study is the threshold for indicating likely experiencing high professional fulfillment. The percentage of participants who endorsed professional fulfillment, with a score of 18 or higher increased from 27.4% (*N* = 55) to 68.2% (*N* = 137). This indicates a large effect size for this improvement in professional fulfillment (Cohen’s *d* 0.95).

There was significant improvement in all the scores related to each question within this category of questions (*p* < 0.001) (Table 1). These included improvement in feeling happy at work, feeling worthwhile at work, feeling satisfied with work, feeling in control when dealing with difficult problems at work, feeling that work is meaningful and contributing professionally in ways that they most value.

### Burnout

A score of 14 or higher indicates a person is likely experiencing burnout. At baseline 77.1% (*N* = 155) of participants endorsed burnout. Mean burnout scores decreased from 20.52 (SD of 9.0), to 11.14 (SD 7.89) (*p* < 0.001) after the intervention. After the program completion, 33.3% (*N* = 67) of these same participants met criteria for burnout (*p* < 0.001), with a 43.8% reduction in burnout overall. This change indicates a large effect size for this change in burnout (Cohen’s *d* 1.11).

### Self-valuation (compassionate self-improvement perspective)

Respondents with a score greater or equal to a score of 9 are more likely to have a compassionate self-improvement perspective. At baseline 17.9% (*N* = 36) of the participants in this study endorsed a compassionate self-improvement perspective compared with 64% eight weeks later (*p* < 0.001). Self-valuation scores improved from 5.79 (SD of 3.16) at baseline to 9.43 (SD of 2.56) (*p* < 0.001). There was a very large effect size for this in compassionate self-improvement (Cohen’s *d* 1.28).

### Program assessment and potential public health impact

The reach, effectiveness, adoption, implementation, and maintenance of this program were analyzed using the RE-AIM framework summarized in (Table 2).

**Table 2:** Analysis of an 8-week virtual coaching program for women physicians using the Reach, Effectiveness, Aim, Implementation, and Maintenance (RE-AIM) framework. June 2020 – November 2021. *N* = 201

Reach, Effectiveness, Aim, Implementation, and Maintenance (RE-AIM) element	Outcome
Reach	Exclusion criteria: Must be MD or DO
Effectiveness	Primary outcomes: Large effect size on professional fulfillment and burnout and very large effect size on self-valuation (self-compassion)
Adoption	Setting exclusions: none Settings approached who implemented: individual level implementation Characteristics of settings: highly variable Utilization: 97% completed sessions
Implementation	Percent of perfect delivery, adaptations: Delivered as intended, no adaptations needed Cost: $5,000-$10,000 per participant Consistency across staff, settings, subgroups: no known inconsistencies
Maintenance	Long term attrition: no significant attrition during the 8-week interventions If program is still ongoing 6 months after study: Yes, the same program is still being delivered over 2 years after the conclusion of the study period. If and how adopted long term: Implemented long term with minimal variation. Many clients went on to train as physician coaches themselves which contributes to future potential broader reach and implementation of physician coaching Alignment of organization mission or sustainability: mission statements of EWP coaching company are well-aligned with addressing physician well-being. The organization is committed to the sustainability of this program.

## Discussion

This study found that a virtual coaching program for women physicians improved measures of well-being (self-compassion and professional fulfillment) and decreased burnout. To the authors’ knowledge, the effect size of this community-based intervention is higher than any previously published interventions in physician well-being [17, 31]. The ability to observe a large effect size on measures of physician distress during the two years of the COVID-19 pandemic is noteworthy and encouraging given the challenging time period, particularly in a group that reported 77% burnout at baseline. The very large effect size (cohen’s d = 1.27) seen with self-valuation/self-compassion was striking, as this measure has historically been one of the most challenging to impact [30, 32]. Improved self-valuation scores have been associated with decreased suicidal ideation [20]. 

Study after study has documented burnout in physicians for decades. Virtual coaching programs offered by independent entities are a viable and effective option to begin to address physician distress. Physicians do not need to wait until their organization offers coaching to access this effective intervention. A broad range of coaching interventions are needed to address physician personal and professional well-being. This particular coaching intervention combined large group recorded calls, as well as small group and private 1:1 session that were not recorded. This multipronged approach accommodated various needs for privacy, while facilitating community and normalization of the common issues faced by women physicians.

With the current crisis of physician burnout affecting both the well-being of physicians and patients, coaching is a promising intervention [16, 17, 19]. Physicians express a growing concern over lack of autonomy in the current healthcare climate [21]. Coaching can help counteract some of the learned helplessness and start to help them identify areas in which they do have choice, agency, and self-efficacy. While coaching is an individual level intervention, an additional benefit is to support the people who then help change the system. Individual solution does not remove the need for concurrent system level changes.

Some physicians perceive that over time, the medical system has been able to exploit the altruism that brought us to this profession. However, this is not sustainable. In order to avoid a public health crisis and significant exodus from medicine in the coming years, physicians must find a way to practice medicine in a sustainable fashion that makes room for and encourages personal and professional well-being [2]. The traditional ways of continually asking physicians to give more than they are comfortable giving cannot continue. It is perhaps the most important work of our generation of physicians to allow physicians to create working conditions that are sustainable and may vary in different stages of life.

​​To the authors’ knowledge, this is the first published data of the effectiveness of widely available coaching for physicians during the COVID pandemic, which has accelerated burnout symptoms in a large population of physicians. It is also the largest and farthest-reaching intervention documented in attending physicians to date. This study suggests that the shared understanding of medical training and medical practice may be particularly impactful. The availability of an external physician coach may also play an important role in creating psychological safety.

This study has several limitations including self-selection bias and lack of randomization. Additionally, the intensity of this intervention is higher than is typically reported in the literature. This intervention was provided by one company; however, it was delivered by over 40 physician coaches from across the United States and Canada, from various specialties and backgrounds, which supports generalizability.

Future research is needed to examine which elements of coaching programs are most impactful, the dose effect of coaching, the lasting effect of coaching throughout the professional life span of physicians, and the influence of certified physician coaches. Additional studies could be designed to examine if there are any meaningful differences in coaching preferences or impact by gender. Further exploration is needed to determine how to best tailor coaching interventions to people of various backgrounds. Race, in addition to gender, may impact a physician’s response to coaching [33] and attention must be paid to the unique needs of physicians with marginalized identities.

In addition, the economics of implementing coaching programs must be considered. The cost of physician burnout has been estimated at $4.6 billion annually by way of reduced clinical hours and high turnover [26]. A comparison of this estimate, which may be low as it was calculated before the COVID-19 pandemic and the subsequent increases in burnout, to the cost of large-scale physician coaching programs is warranted.

Although coaching is common in business and executive leadership, to date it has been made available in medicine often only for a small number of leaders or for distressed physicians. However, there is a growing movement to consider that every physician deserves a coach, just as every athlete receives a coach. This study further illustrates the impact of coaching on personal and professional wellbeing and adds new evidence to support the broader use of coaching for physicians.

### Electronic supplementary material

Below is the link to the electronic supplementary material.

Supplementary Material 1

## Data Availability

The datasets during and/or analyzed during the current study available from the corresponding author on reasonable request.
