# Supplementary material for: Impact of a virtual coaching program for women physicians on burnout, fulfillment, and self-valuation
Source: BMC Psychol. 2024 Jun 5;12:331. doi: 10.1186/s40359-024-01763-0 (PMC11155151; doi:10.1186/s40359-024-01763-0)
Supplement: Supplementary file 1 — Supplementary Material 1 [file 40359_2024_1763_MOESM1_ESM.docx]

Appendix A.  Components of the Empowering Women Physicians Virtual Coaching Program Components June 2020 – November 2021.

| **Coaching program components available to each participant** |  |
| --- | --- |
| One on one private coaching sessions (50 minutes) | 8 |
| Small group coaching sessions (90-120 minutes+) | 6 |
| Large group coaching sessions (120 minutes +) | 24 |
| Life coaching | 12 |
| Business coaching | 8 |
| Visiting guest expert workshop (non physicians) | 4 |
| **Recorded coaching call library** | Available on demand via password protected web portal |
| **Access to live coaching calls** | Live calls from current session are available on via password protected website and private podcast |
| **Number of live hours available per program minimum** | 65 |
| **Example of scheduled group coaching topics** | Charting, parenting, habits, marriage, relationships, general physician personal and professional coaching sessions |
| **Coaches** | All one on one and group coaching sessions (except for visiting guest expert workshops) are conducted by female physicians who are certified coaches. |
| **Program cost** | $5,000 to $10,000 USD |
